# Experiences of long-term life-limiting conditions among patients and carers: what can we learn from a meta-review of systematic reviews of qualitative studies of chronic heart failure, chronic obstructive pulmonary disease and chronic kidney disease?

**DOI:** 10.1136/bmjopen-2016-011694

**Published:** 2016-10-05

**Authors:** Carl R May, Amanda Cummings, Michelle Myall, Jonathan Harvey, Catherine Pope, Peter Griffiths, Paul Roderick, Mick Arber, Kasey Boehmer, Frances S Mair, Alison Richardson

**Affiliations:** 1 Faculty of Health Sciences, University of Southampton, Southampton, UK; 2 NIHR CLAHRC Wessex, University of Southampton, Southampton, UK; 3 University Hospital Southampton NHS Foundation Trust, Southampton, UK; 4 Faculty of Medicine, University of Southampton, Southampton General Hospital, Southampton, UK; 5 York Health Economics Consortium, University of York, York, UK; 6 Knowledge and Evaluation Research Unit, Mayo Clinic, Rochester, Minnesota, USA; 7 Department of General Practice and Primary Care, Institute of Health and Wellbeing, University of Glasgow, Glasgow, UK

**Keywords:** long-term conditions, patient experience, burden of treatment, end of life, meta-review

## Abstract

**Objectives:**

To summarise and synthesise published qualitative studies to characterise factors that shape patient and caregiver experiences of chronic heart failure (CHF), chronic obstructive pulmonary disease (COPD) and chronic kidney disease (CKD).

**Design:**

Meta-review of qualitative systematic reviews and metasyntheses. Papers analysed using content analysis.

**Data sources:**

CINAHL, EMBASE, MEDLINE, PsychINFO, Scopus and Web of Science were searched from January 2000 to April 2015.

**Eligibility criteria for selecting studies:**

Systematic reviews and qualitative metasyntheses where the participants were patients, caregivers and which described experiences of care for CHF, COPD and CKD in primary and secondary care who were aged ≥18 years.

**Results:**

Searches identified 5420 articles, 53 of which met inclusion criteria. Reviews showed that patients' and caregivers' help seeking and decision-making were shaped by their degree of structural advantage (socioeconomic status, spatial location, health service quality); their degree of interactional advantage (cognitive advantage, affective state and interaction quality) and their degree of structural resilience (adaptation to adversity, competence in managing care and caregiver response to demands).

**Conclusions:**

To the best of our knowledge, this is the first synthesis of qualitative systematic reviews in the field. An important outcome of this overview is an emphasis on what patients and caregivers value and on attributes of healthcare systems, relationships and practices that affect the distressing effects and consequences of pathophysiological deterioration in CHF, COPD and CKD. Interventions that seek to empower individual patients may have limited effectiveness for those who are most affected by the combined weight of structural, relational and practical disadvantage identified in this overview. We identify potential targets for interventions that could address these disadvantages.

**Systematic review registration number:**

PROSPERO CRD42014014547.

**Strengths and limitations of this study**

- This is the first synthesis of qualitative systematic reviews focusing on patient and carer experience of life-limiting chronic conditions that consider them against pathophysiological deterioration towards the terminal phase of illness.
- The review builds on systematic review and analysis to develop a robust conceptual model of the factors that shape patient and caregiver expectations and choices about help seeking and self-care, and which shows how these are the products of rational decisions and experiential processes.
- The review demonstrates the value of qualitative research that identifies and characterises important aspects of patient experience and health and healthcare-related behaviours.
- The review provides proposed domains of patient and caregiver experience that may represent potential targets for new interventions to support patients and caregivers to improve capacity and better manage workload to promote improved experience of illness.
- This is an overview of a heterogeneous set of papers and as such there was considerable variability in research aims, methods and perspectives which is a limitation of the study.

## Introduction

Globally, health services are responding to an increasingly older population characterised by complexes of multimorbidity that include long-term life-limiting conditions.^1^ As the burden of disease grows, so too does another kind of problem—burden of treatment^2^—which occurs as the work of disease management has been shifted from formal healthcare provision to self-management at home. This shift has been a major focus of policy effort in health services that deliver care for people with long-term conditions.^3^ ^4^ However, for a significant proportion of people with chronic and often life-limiting conditions, illness trajectories are characterised by major disruptions.^5^ ^6^ They include increasingly frequent cycles of hospital admission and discharge, supported self-management at home and readmission as exacerbation events occur.^7^ Here, there is a complex balancing act to be performed around the work of being a patient. This involves managing the limiting effects of symptoms while also managing complex therapeutic regimens, self-monitoring technologies and assessment regimes, and interactions with healthcare providers and organisations.^8–10^ These admission–discharge–readmission cycles may lead to increasing experiences of complex workload for patients and their carers which can sometimes be overwhelming and may have important effects of quality of life.^2^ ^11–13^

Understanding the parameters of patient workload and the experiences of complexity that stem from it has recently become an important focus of research on long-term conditions,^14–20^ and has led to modelling work that has focused on the relationships between treatment burden, symptom burden and healthcare systems.^12^ ^13^ ^21–23^ This has taken place against the background of a programme of policy and practice development that, internationally, focuses on reworking the sick-role and rethinking the relationship between the sick person and healthcare system,^24^ and understanding the dynamic role of social networks in supporting them.^25^ Yet problems remain in the way that researchers, clinicians and policymakers understand patients with long-term conditions. In policy terms, this group is sometimes seen as the source of inappropriate and excess demand on primary care services and emergency departments.^26^ These factors are becoming increasingly important as healthcare systems find themselves under significant pressure to control costs and reduce spending.

Much clinical and health services research on long-term conditions focuses on controlling symptoms or delaying their onset—achieving equilibrium, even when this may be punctuated by acute exacerbation events—but in this paper, we are concerned with the experiences of people with chronic heart failure (CHF), chronic obstructive pulmonary disease (COPD) and chronic kidney disease (CKD) as they approach the terminal phase of their illness. Here, factors leading to pathophysiological deterioration limit their capacity to participate independently in self-management and healthcare processes.^27^ As this happens, they experience new dependencies on health services and new demands on informal networks that provide care and social support. All of these factors must be balanced against the wider demands of everyday life,^23^ and we need to better understand how these problems are framed and experienced by people with long-term conditions, and this review aims to ‘identify, characterise and explain the common factors that shape patient journeys through care in CHF, COPD and CKD’^28^ in order to inform future intervention development.

## Methods and analysis

### Eligibility criteria

This is a systematic review including data from qualitative reviews and the eligibility criteria for study inclusion have been developed using the PICO (participants, interventions, comparators and outcomes) framework (box 1).

Box 1 **PICO criteria for including studies**

▸ Population: Patients (aged >18 years and diagnosed with chronic heart failure, chronic kidney disease or chronic obstructive pulmonary disease), and formal or informal caregivers and health professionals in healthcare settings (including triage services, emergency departments, in-patient hospital care, outpatient/ambulatory care departments, primary care service/family practice doctor's offices, community nursing services or at home).

▸ Intervention: Experiences of healthcare provision.

▸ Comparator: This review not limited to comparator studies. Where comparators are present these may include: usual care or control groups.

▸ Outcomes: Qualitative data on patients and caregivers experiences of care for those with chronic heart failure, chronic obstructive pulmonary disease or chronic kidney disease.

▸ Study type: Secondary studies (qualitative or mixed method systematic reviews, qualitative meta-syntheses and meta-ethnographies).

### Inclusion and exclusion criteria

We included papers that met the PICO criteria (box 1) and were published in English reporting qualitative reviews of patients' or caregivers' experiences of healthcare provision. Some of these studies also included the views of health professionals. Mixed methods reviews were included if analysis of primary qualitative studies could be clearly differentiated from analysis of other kinds of primary studies in the text. Papers were excluded if they were: reports of treatment; reports of healthcare organisation or delivery which were not concerned with patients' or caregivers' experience; summaries or discussions of the literature or editorials, notes, letters and case reports.

### Searches and information sources

**Figure 1:**
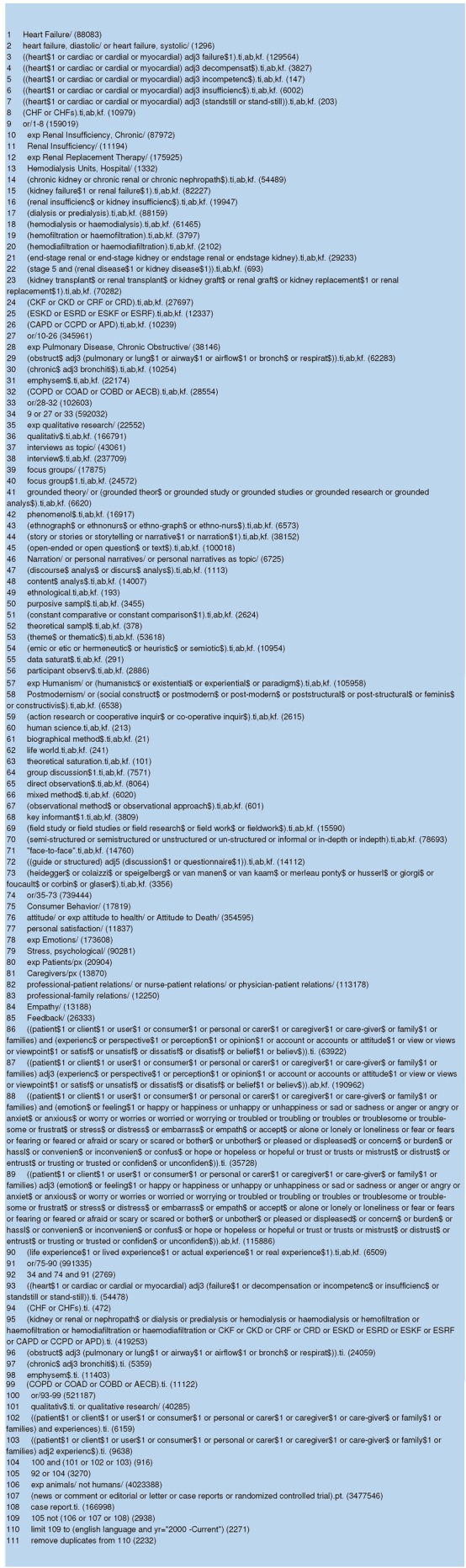
Search strategy.

Searches were conducted in the following bibliographic databases: MEDLINE(R) In-Process and Other Non-Indexed Citations and MEDLINE(R); Embase; CINAHL Plus; Science Citation Index Expanded; Social Sciences Citation Index; Arts and Humanities Citation Index; PsycINFO; and Scopus. Searches were completed by April 2015 and identified papers published between 1 January 2000 and 31 December 2014. Full details of the search strategy are provided in figure 1.

### Study selection and data extraction

All titles and abstracts were screened independently by AC and CRM or AC and MM. Screening of full-text articles was undertaken independently by AC and CRM with assistance from JH. Disagreements about eligibility for inclusion at title and abstract screening stage were resolved by discussion and majority vote; disagreements about inclusion at full-paper screening were resolved by discussion, or by referral (n=3) to colleagues not involved in this review.

### Quality assessment

Although there are now several quality assurance frameworks for assessing primary qualitative studies,^29–31^ there are none that are generally accepted for meta-reviews of such studies. Quality appraisal of included papers was conducted retrospectively at the request of the journal, and was undertaken using a tool that combined elements of the AMSTAR^32^ and CASP^33^ ^34^ instruments. The tool is shown in online supplementary appendix 1. Each paper was appraised independently by two researchers. Overall scores were expressed as a percentage. Papers scoring over 80% were awarded a high (H) quality rating, those between 60% and 80% a medium (M) rating and papers scoring below 60% were attributed a low (L) rating. No papers were excluded on grounds of quality, and agreement statistics were not calculated. Results of this exercise (presented in tables 1–3) should be treated with considerable caution because this meta-review includes studies using integrative (mixed methods) reviews, thematic analyses and qualitative metasyntheses applied in very different ways by their authors.

**Table 1:** Summary of overview of qualitative systematic reviews and metasyntheses of experiences of CHF

Review	Year	Type of review	Phenomena of interest	Number of qualitative studies included	Key aim(s)	Quality score
Molloy *et al*^38^ (UK)	2005	Mixed methods (integrative) review	Role of family caregivers	3/16	Evaluate effect of CHF on caregivers' well-being; evaluate role of caregivers in management of CHF; outline policy and practice implications of current studies	77% M
Yu^39^	2007	Qualitative systematic review with thematic analysis of qualitative studies	Older people's experiences	14	Synthesising qualitative studies of people with CHF to advance understanding of lived experience and inform intervention and service development	94% H
McEntee *et al*^40^ (USA)	2009	Mixed methods (integrative) review	Barriers to CHF care	15	To synthesise research on barriers to CHF care at patient, provider and system levels	61% M
Westland *et al*^41^ (UK)	2009	Qualitative integrative review of living with heart failure	Experiences and perceptions of people living with CHF	18	To explore the experiences and perceptions of patients who have heart failure	M 77%
Hopp *et al*^42^ (USA)	2010	Systematic review with thematic analysis of qualitative studies	Older people's experiences	15	Understand the ‘lived experience’ of CHF among older people to inform social work practice with this group	72% M
Jeon *et al*^43^ (Australia)	2010	Narrative review	Experience of living with CHF	30	Conduct a narrative review of qualitative studies of people's experiences of living with CHF to develop a wide-ranging understanding of the patient experience of CHF	M 77%
Barclay *et al*^44^ (UK)	2011	Systematic review with narrative synthesis of qualitative studies	End-of-life care in CHF	17/23	Identify patient and professional preferences around end-of-life conversations in CHF, and to identify barriers and facilitators of these conversations	77% M
Dev *et al*^45^ (USA)	2011	Qualitative metasynthesis	Self-care CHF with comorbid conditions	3	Identify and characterise factors that affect integration of CHF self-care with other comorbid conditions	38% L
Dickson *et al*^46^ (USA)	2011	Systematic review with thematic analysis of qualitative studies	Self-care in CHF with comorbidities	3	Identify and characterise aspects of self-care for CHF that are complicated by comorbidities	55% L
Kang *et al*^47^ (China)	2011	Qualitative metasynthesis	Role of family caregivers	10	Synthesise qualitative studies of caregivers’ experiences of CHF to inform nurses as they support families affected by CHF	94% H
Low *et al*^48^ (UK)	2011	Systematic review with thematic analysis of qualitative studies	End-of-life care	28/48	Explore studies on patient and professional understandings of disease processes and perceived needs and experiences of care provision in palliative care for CHF	77% M
Tierney *et al*^49^ (UK)	2011	Qualitative systematic review with framework analysis	Physical activity	20	Identify barriers and facilitators of physical activity in CHF and identify beliefs and behaviours that could be targeted by interventions to promote activity	88% H
Thomas and Clark^50^ (Canada)	2011	Qualitative metasynthesis	Sex and gender	5/6	Identify and understand sex-related and gender-related factors that shape women's self-care beliefs and behaviours in CHF	83% H
Clark *et al*^51^ (Canada)	2012	Qualitative metasynthesis	Help-seeking decisions and behaviours	58	Identify and characterise elements of help seeking in CHF and model the main factors and processes associated with help-seeking decisions	94% H
Jani *et al*^52^ (UK)	2012	Qualitative systematic review with framework analysis	Treatment burden in heart failure at end of life	16	Identify, characterise and explain workload associated with treatment burden in CHF	83% H
Procter^53^ (UK)	2012	Qualitative systematic review with thematic analysis of qualitative studies	Contribution of palliative care specialists to end of life care	5	Identify barriers to collaborative working in palliative care, and characterise CHF patient and carer expectations and needs	83% H
Rolls and Young^54^ (USA)	2012	Discourse analysis	CHF from the perspective of older women	4	From a feminist standpoint, to critically apprehend women's lived experiences of CHF from the perspective of women themselves	77% M
Buck *et al*^55^ (Canada)	2013	Systematic review with content analysis of qualitative studies	Caregivers’ contributions to self-care	13/30	Identify and characterise specific activities by which caregivers contribute to self-care beliefs and behaviours in CHF	88% H
Falk *et al*^56^ (Sweden)	2013	Mixed methods (integrative) review	Lived experience of CHF among older patients	5/23	Synthesise knowledge about self-reported symptoms, illness experience and self-care management by older patients with CHF	77% M
Siabani *et al*^57^ (Australia)	2013	Qualitative metasynthesis	Factors that promote or inhibit self-care	23	Identify the factors that prevent optimal engagement with self-care regimens to inform future intervention development	77% M
Sookhoo *et al*^58^ (UK)	2013	Qualitative metasynthesis	Participation in CHF self-management education programmes	8	Synthesise studies about experiences of educational interventions to support people with CHF in self-care	83% H
Clark *et al*^59^ (Canada)	2014	Qualitative metasynthesis	Patients and caregivers’ perceptions of effective self-care	49	Synthesise studies about the determinants of effective self-care in CHF, identify and characterising experiences	83% H
Dekker^60^ (USA)	2014	Qualitative systematic review with thematic analysis of qualitative studies	Experiences of depressive symptoms	13	Identify and characterise the contributing factors, role and effects of depressive symptoms in shaping experiences of CHF	44% L
Harkness *et al*^61^ (Canada)	2014	Qualitative metasynthesis	Strategies for self-care	47	Identify strategies used by people with CHF to accommodate self-care techniques in daily life	83% H
Strachan *et al*^62^ (Canada)	2014	Qualitative metasynthesis	Contextual factors that influence self-care	45	Identify and characterise elements of social context that affect self-care in CHF	83% H
Wingham *et al*^63^ (UK)	2014	Meta-ethnography	Attitudes, beliefs, expectations and experiences of self-management	19	Develop an explanatory model of patients’ attitudes, beliefs, expectations and experiences of self-management in CHF that could inform the development of future self-management strategies	88% H

CHF, chronic heart failure.

**Table 2:** Summary of overview of qualitative systematic reviews and metasyntheses of experiences of COPD

Review	Year	Type of review	Phenomena of interest	Number of qualitative studies included	Key aim(s)	Quality score
Gysels *et al*^64^ (UK)	2007	Qualitative systematic review with thematic analysis of qualitative studies	Experiences of breathlessness	22	Synthesise qualitative evidence about breathlessness as a common symptom of multiple conditions, including COPD (and CHF)	83% H
Cullen and Stiffler^65^ (USA)	2009	Qualitative metasynthesis	Experiences of oxygen therapy	4	Identify and characterise new evidence about patients’ perspectives on oxygen therapy in COPD and other conditions to guide intervention development	83% H
Disler *et al*^66^ (Australia)	2011	Mixed methods (integrative) review	Factors that promote or inhibit self-care	13/44	Identify and characterise factors that influence self-care in COPD	88% H
Keating *et al*^67^ (Australia)	2011	Mixed methods (integrative) review	Barriers to participation in rehabilitation	5/11	Identify and characterise factors that affect attendance and participation in pulmonary rehabilitation for COPD	88% H
Kirkpatrick *et al*^68^ (UK)	2012	Qualitative metasynthesis	Patients and caregivers’ perceptions of support	39	Identify and characterise forms and experiences of support for people with COPD from informal and formal caregivers, and identify and characterise those forms of support believed to be beneficial	77% M
Giacomini *et al*^69^ (Canada)	2012	Qualitative metasynthesis	Experiences of living and dying with COPD	101	Synthesise studies of people with COPD and their informal caregivers, identify and characterise insights into their experiences and inform future interventions	88% H
Thorpe *et al*^70^ (Australia)	2012	Mixed methods (integrative) review	Participation in physical activity	8/11	Identify and characterise barriers and enablers of participation in physical activity, including pulmonary rehabilitation, among people with COPD	94% H
Langer *et al*^71^ (UK)	2012	Qualitative metasynthesis	Use of unscheduled/emergency care in long-term conditions	5/42	Identify and characterise the range of psychosocial and other influences on use of unscheduled care by people with long-term conditions, including COPD and CHF	83% H
Momen *et al*^72^ UK	2012	Qualitative systematic literature review and narrative synthesis	End-of-life conversations	12/30	Identify factors that promote or inhibit discussions of management of end of life in COPD	66% M
De Souza Pinto *et al*^73^ (Spain)	2013	Meta-ethnography	Impact of pulmonary rehabilitation	8	Explore the lived experience of COPD and identifying positive and negative aspects of pulmonary rehabilitation from the perspective of people with COPD	94% H
Harrison *et al*^74^ (UK)	2013	Qualitative metasynthesis	Impact of acute exacerbation events in COPD	8	Identify and characterise people's experiences of exacerbation events that inhibit engagement with pulmonary rehabilitation in COPD	100% H
Disler *et al*^75^ (Australia)	2014	Qualitative metasynthesis	Lived experience of COPD	22	Identify and characterise the lived experience of COPD and inform the development of healthcare services	83% H
Oishi *et al*^76^ (UK)	2014	Mixed methods (integrative) review	Provision of palliative care for patients with non-cancer	17/30	Identify and characterise aspects of professional role, performance and barriers and facilitators to primary palliative care	77% M

CHF, chronic heart failure; COPD, chronic obstructive pulmonary disease.

**Table 3:** Summary of overview of qualitative systematic reviews and metasyntheses of experiences of CKD

Review	Year	Type of review	Phenomena of interest	Number of qualitative studies included	Key aim(s)	Quality score
Low *et al*^77^ (UK)	2008	Mixed methods (integrative) review	Impact of end-stage CKD on caregivers	11/36	Identify all studies exploring the impact of CKD on ‘close persons’ during and after withdrawal from dialysis, and during end-of-life care	77% M
Morton *et al*^78^ (Australia)	2010	Qualitative systematic review with thematic analysis of studies	Views of people and carers on treatment decision-making in CKD	18	Identify patient and carer perspectives on treatment decisions, and characterise factors that shape these decisions	77% M
Wadd *et al*^79^ (Australia)	2011	Qualitative systematic review with thematic analysis of studies	Parents’ experiences of dialysis for CKD	17	Identify and characterise experiences of parenting while undergoing dialysis for CKD, to inform family-centred holistic care	72% M
Bayhakki and Hattakit^80^ (Indonesia)	2012	Qualitative metasynthesis	Experience of haemodialysis for CKD	10	Characterise and understand aspects of lived experience of haemodialysis	83% H
Harwood and Clark^81^ (Canada)	2012	Qualitative metasynthesis	Decision-making about treatment modalities in CKD	16	Understand how people with CKD decide on treatment modalities and to explain underuse of home dialysis	83% H
Makaroff^82^ (Canada)	2012	Qualitative metasynthesis	Lived experience of end-stage CKD	13	Understand experiences of people with end-stage CKD aiming to inform future interventions	77% M
Moustakas *et al*^83^ (Australia)	2012	Mixed methods (integrative) review	Supportive care needs of older people with advanced CKD	4/12	Identify and characterise supportive care needs of older people with advanced CKD, to inform future research and intervention design	66% M
Tong *et al*^84^ (Australia)	2013	Qualitative systematic review with thematic analysis of studies	Perspectives of adults living with peritoneal dialysis	39	Identify and characterise experiences, attitudes and beliefs relating to peritoneal dialysis	83% H
Casey *et al*^85^ (Australia)	2014	Qualitative systematic review with thematic analysis of studies	Perspectives on haemodialysis vascular access	46	Identify and characterise the psychosocial impact of vascular access for haemodialysis, and the concerns, beliefs and attitudes of patients during treatment. Informing strategies to maximise quality of life and quality of care	88% H
Luckett *et al*^86^ (Australia)	2014	Mixed methods (integrative) review	Advance care planning for adults	6/54	Identification of advance care planning interventions and evaluations and inform understanding of barriers and facilitators to implementation	100% H
Palmer *et al*^87^ (New Zealand)	2014	Qualitative systematic review with thematic analysis of studies	Patients view of dietary and fluid restrictions in CKD	46	How people with CKD meet the challenge of managing complex fluid and dietary requirements, to inform clinical practice	83% H
Tong *et al*^88^ (Australia)	2014	Qualitative systematic review with thematic analysis of studies	Women's experiences of pregnancy and CKD	15	Identify and characterise factors that affect how women with CKD experience pregnancy, to inform shared decision-making processes and to optimise quality of care and quality of life	83% H
Tong *et al*^89^ (Australia)	2014	Qualitative systematic review with thematic analysis of studies	Patient and caregiver perspectives on end-of-life care	26	Synthesising patients and caregiver views on decisions to initiate or withdraw from dialysis and for end-of-life care in CKD, to inform strategies to optimise quality of care and quality of life	83% H
Walker *et al*^90^ (Australia)	2014	Qualitative systematic review with thematic analysis of studies	Patient and caregiver perspectives on home dialysis	24	Describe patient and caregiver experiences of home dialysis to inform the development of service interventions	83% H

CKD, chronic kidney disease.

10.1136/bmjopen-2016-011694.supp1 supplementary appendix

### Data analysis and synthesis

Formal data for analysis consisted of the discussion and conclusions sections of each included paper, and a qualitative content analysis^35^ ^36^ of attributions within these was undertaken. An attribution is a statement that characterises a state and that relies on a causal inference or explanation about the supposed antecedents of that state.^37^ Attributions were identified on (1) patient and caregivers' experiences of illness and journeys through care, (2) experiences of healthcare practices and (3) evaluations of illness and healthcare practices. Each identified attribution was matched to any causal inferences and explanations for it that were made by authors. Where such attributions were unexplained, and where explanations could not be directly linked to an attribution about patient or caregiver experience, they were traced back through the analysis presented in the results section of each review or metasynthesis. This tracing work was especially important where integrative reviews were concerned since these drew on quantitative and qualitative primary studies and we wished to exclude the former from our analysis. Related attributions and explanations were grouped together in sets and then simple explanatory propositions were formulated to characterise them. Formal analysis of textual data was undertaken by CRM and then reviewed in detail by other authors.

## Results

### Results of searches

**Figure 2:**
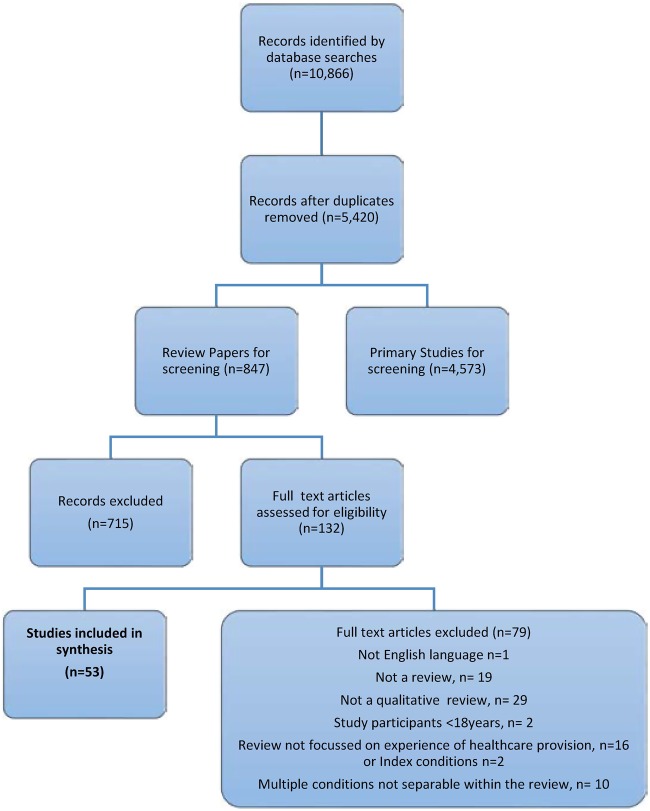
PRISMA flow chart.

The review process is shown in figure 2: 10 866 possible articles were identified, and after the removal of duplicates, 5420 were left. Of these, 847 were reviews or metasyntheses. Further screening identified 132 potentially eligible papers and full-paper reading led to 53 reviews that fully met the criteria for inclusion. These reviews consisted of 26 articles concerned with CHF^38–63^ (see table 1); 13 articles concerned with COPD^64–76^ (see table 2) and 14 studies of CKD^77–90^ (see table 3). Of these, most reviews focused on aspects of everyday experiences of long-term conditions with an emphasis on understanding patient and caregiver behaviours related to self-management regimens, and a subset of 10 papers explored elements of experiences of care towards end of life.^44^ ^48^ ^52^ ^53^ ^69^ ^76^ ^77^ ^82^ ^86^ ^89^ These 53 reviews synthesised the results of 559 reports of primary qualitative studies.

Three types of review method are represented in this sample of papers: integrative reviews that synthesised qualitative studies alongside other studies (often cross-sectional surveys, reviews of routinely collected clinical data and case notes);^38^ ^40^ ^56^ ^66^ ^67^ ^70^ ^76^ ^77^ ^83^ ^86^ systematic reviews of qualitative studies that employed some kind of thematic analysis;^39^ ^41–44^ ^46^ ^48^ ^49^ ^52^ ^53^ ^55^ ^60^ ^64^ ^72^ ^78^ ^79^ ^84^ ^85^ ^87^ ^88^ ^90^ and reviews that were identified by their authors as qualitative meta-syntheses or meta-ethnographies.^45^ ^47^ ^50^ ^51^ ^57–59^ ^61–63^ ^65^ ^68^ ^69^ ^71^ ^73–75^ ^80–82^ ^89^ In addition, one paper was explicitly characterised as a discourse analysis by its authors.^54^ Irrespective of the method that authors stated that they had employed, the body of reviews and metasyntheses included in this synthesis were largely descriptive in their approach to data analysis.

### Social structure

Socioeconomic disadvantages that play a role in structuring the unequal distribution of health problems and inequalities in access to health services were often treated as ‘contextual’ factors,^49^ ^62^ ^70^ ^71^ ^73^ ^76^ and reviews emphasised the importance of social inequalities, employing broad and inclusive definitions of disadvantage that include access to transport and other socioeconomic resources, and environmental factors such as poor air quality and exposure to other sources of environmental pollution. Other structural variables, such as gender and ethnicity, were not well represented at all.^42^ ^50^ ^54^ ^88^ One demographic variable that was given consideration was age: many reviews explicitly dealt with CHF, COPD and, to a lesser extent, CKD as diseases of old age. The views of patients and caregivers were often that such diseases were a ‘natural’ consequence of ageing.

### System behaviour

Experiences of poorly coordinated and organised care,^43^ ^45^ ^48^ ^52^ poor communications between professionals^40^ ^45^ ^47^ ^48^ ^52^ and between patients and professionals^42^ ^47^ were common. Even so, patients and caregivers had high expectations of clinicians, especially when they needed urgent assistance,^76^ even though clinicians were sometimes seen as lacking important expertise around coordinating care in multimorbidities.^46^ Treatment decisions were restricted not only by the quality and timing of information but also by dealing with the prospect of death.^78^ ^81^ Conversations about treatment decisions were often focused on day-to-day problems of disease management,^44^ ^53^ ^78^ ^83^ and seemed to be rarely oriented towards decisions about the future—especially about palliative and supportive care, and discontinuation of treatment (eg, implantable cardiac devices or dialysis).^45^ ^81–83^ ^86^ Reviews suggested that conversations about end-of-life issues rarely took place, either because of patients' poor understanding of their conditions or because professionals recognise high levels of prognostic uncertainty and the risk of sudden death. When patients were aware of disease progression and potential prognosis, they were able to discuss end-of-life issues openly,^53^ although the key decision-maker was the clinician.^44^ ^48^ ^83^ This may mean that the life-limiting nature of disease was not apparent to patients.^41^ ^75^ When it was, some patients saw living wills, and advance care plans as useful decision-making tools,^77^ even though these could lead to conflict and discomfort.^59^ Experiences of poor communications seemed to be endemic in the patient groups included in all of the reviews included in this synthesis, and common to all healthcare systems.

### Understanding disease progression and symptoms

Reviews emphasised the importance of patients' and caregivers' poor understanding of disease, disease progression and the significance of symptoms.^40^ ^41^ ^43^ ^45^ ^50^ ^52^ ^58^ ^59^ ^69^ ^75^ ^76^ ^88^ Women and older people were presented as being particularly vulnerable to a lack of correct knowledge;^39^ ^50^ ^54^ and most patients were seen as ill-prepared to face their disease^45^ and symptoms.^88^ Symptom recognition was particularly challenging, in the context of comorbidity, for patients, caregivers and clinicians.^43^ ^45^ ^57^ Poor understanding of disease mechanisms and progression was characterised as a key source of distress for patients and caregivers.^64^ It was sometimes attributed to low levels of health literacy^56^ and to fragmented, limited and ineffective educational resources.^57^ ^83^ It led to negative consequences that included failure to understand the life-limiting nature of disease; wrong beliefs about the causes of acute exacerbations of symptoms; incorrect assumptions and actions that follow from these;^41^ ^46^ ^64^ ^69^ feelings of powerlessness over disease progression^74^ and low expectations of clinical interventions.^76^ These factors were seen to interfere with symptom management and help seeking,^65^ ^66^ and adherence to self-management regimens.^57^ ^61^ They were especially important where patients needed to understand how comorbidities ‘fitted’ with each other^46^ ^70^ and needed to manage complex polypharmacy.^64^ Unpredictable disease progression made planning daily and longer term activities difficult and either meant that threat to life was not explicitly acknowledged, or that if it was, uncertainty interfered with planning for end of life and the initiation of palliative care.^39^ ^42^ ^76^ ^82^

### Self-management regimes

Self-management regimes include participating in pulmonary and cardiac rehabilitation programmes; adhering to instructions about exercise and diet; adhering to often complex therapeutic regimens; operating and monitoring medical devices (such as implantable cardiac devices, or home oxygen equipment) along with other technologies and assessment regimes. Self-management also includes significant administrative and organisational work, as people with long-term conditions interact with healthcare providers and organisations.^8–10^ Reviews suggested that the performance of self-management tasks was valued by patients, who actively invested in them and displayed considerable resilience as they did so.^58^ ^61^ ^73^ ^84^ They sought to overcome the disruptive effect of illness on their lives and to assimilate self-management strategies into everyday life.^63^ However, negative changes in self-identity, self-esteem, social functioning, physical capacity and experiences of social loss were common.^41^ ^43^ ^79^ ^84^ Fear, anxiety, isolation and discomfort were consistently characterised as consequences of disease^49^ ^57^ ^60^ ^82^ that interfered with participation in self-management.^77^ Experienced symptoms and physical limitations led to attempts to avoid physical activities^53^ ^71^ ^80^ in case they precipitated (potentially fatal) acute episodes.^42^ ^47^ ^57^ ^71^ ^74^ Adherence to self-management programmes was adversely affected by disruption to everyday activities and routines,^40^ ^67^ and by conflicts with competing self-management regimes or treatment regimens for comorbidities.^43^ ^45^ ^46^ ^55^ ^58^ ^87^

Reviews also offered evidence of adaptive processes in the face of disease progression and the disruptions that stem from this.^63^ Such adaptations included the accumulation of expertise and associated self-management strategies developed over time.^43^ ^61^ Patients sought to develop strategies that would ensure better symptom control, and that increased the duration of periods between acute episodes and reduced the impact of acute episodes.^67^ ^87^ Poor quality self-management instruction or interactions with health professionals could have adverse effects,^51^ especially because patients were sensitive to evidence of clinicians' doubts about the value of different self-management strategies.^67^

### Burdens for patients and caregivers

Inequalities of access to informal support and material assistance from family and social network members^71^ and health professionals,^40^ ^70^ and problems of continuity of care,^76^ figured prominently across all three conditions. Experiences of symptom burdens (eg, breathlessness, fatigue and anxiety) were characterised as leading to affective responses. These included psychological responses to limitations on everyday activities and relationships that led to social isolation, loss of hope and fear of death.^43^ ^50^ ^66^ ^80^ ^87^ Feelings of worthlessness and burdensomeness^60^ ^89^ were commonly experienced. Some reviews suggested that they were experienced more acutely by women.^41^ ^50^ ^54^ Against this background, social support was characterised as important,^38^ ^84^ especially when it was connected with practical contributions to self-management that might include symptom monitoring and management, information gathering and interaction with health professionals and help seeking at moments of crisis.^55^ Although social support was often marked by shared experiences of solidarity and collective engagement, patients appreciated the disruptive and demanding effects of their illness on caregivers and their wider social networks.^47^ ^81^ ^89^ These included increased workload, and economic consequences—as well as stress, anxiety and isolation—as disease progressed.^38^ ^39^ ^77^ ^90^

Reviews also pointed to the importance of life-sustaining interventions (eg, dialysis and implantable devices) and patient and caregiver preferences for those interventions that promoted self-efficacy and delayed disease progression.^45^ ^73^ They provided opportunities to gain some control and establish a limited degree of normality.^84^ ^90^ However, some patients reported heightened vulnerability, feelings of dependence and unpreparedness, along with anxiety about their capacity to perform procedures and the risks of treatment failure or complications.^85^ ^90^ Using health technologies of different kinds brought only ‘temporary mastery’ over disease, and required continuous attention.^85^ This led to a constant tension between managing disease and technological supports, and the other demands of everyday life.^58^ ^65^ ^79^ Technologies could be intrusive because of the demands that they made on patient and caregiver time and effort.^81^ Assistive technologies could also have the paradoxical effect of leaving patients and caregivers feeling isolated from clinical help and that their homes had been ‘medicalised’.^90^ When technological supports were no longer effective or needed to be withdrawn for other reasons, patients and caregivers often felt abandoned.^81^

### Hospital admissions

Patients valued self-management and care at home, but did not always have full confidence in primary or community care services. They also sometimes found self-management limiting, isolating and frightening.^70^ In this context, help seeking was a problem because of difficulties in recognising and interpreting symptoms.^51^ The security of hospital care is therefore an important element of their experience and they may therefore seek it out.^67^ Experience of acute and uncontrollable exacerbations is frightening and distressing, but patients and caregivers often experience service provision that categorises them as anxious when anxiety is a consequence, rather than a cause, of exacerbation episodes.^64^ Here, patients regarded unscheduled and emergency care as one of a number of sensible options among a range of possible destinations for help seeking.^73^ They therefore regard accessing such care as a rational response to acute disease episodes.^73^

## Discussion

### Overcoming individual deficits

Key features of the literature included in this study are assumptions about the presence and importance of two kinds of deficits. First, there is a patient deficit rooted in poor understanding and non-adherence to treatment regimens, and that is expressed through lack of motivation to participate. Second, there is a professional deficit rooted in poor communications and coordination and that is expressed in reluctance to engage in end-of-life planning. An important finding of our synthesis, however, is that these individual behaviours are linked to structural factors (eg, socioeconomic status, spatial location) and system qualities that are much more than mere ‘context’. They may act as causal mechanisms that have important determining effects on the experiences of patients and caregivers. Their role, however, is underinvestigated. Against the background of these effects, patients and caregivers value resilience, functional performance and social support that make practical contributions to self-management, and they develop cumulative expertise in negotiating self-management tasks and in navigating healthcare systems. They also carry significant burdens. The burden of symptoms—the inevitable consequences of pathophysiological deterioration—may include frightening and potentially lethal acute episodes of disease. The burden of treatment includes additional moral responsibilities, affective and cognitive demands, increased workload and economic consequences of participation in self-management and formal healthcare. Once again, these factors are underinvestigated but may also include mechanisms that likely shape patient and caregiver behaviours.

The effects of pathophysiological deterioration mean that patients and caregivers experience cognitive, affective and interactional disadvantages derived from prognostic uncertainty. They also experience poor communications among health professionals and uncoordinated services, and they have an increasingly limited capacity to make sense of their illness and its effects. Although self-management practices, technological investments and clinical interventions are valued, they may bring only temporary gains. Their benefits are ultimately overwhelmed by disease progression and do not change its final outcome.

### Process tracing model

**Figure 3:**
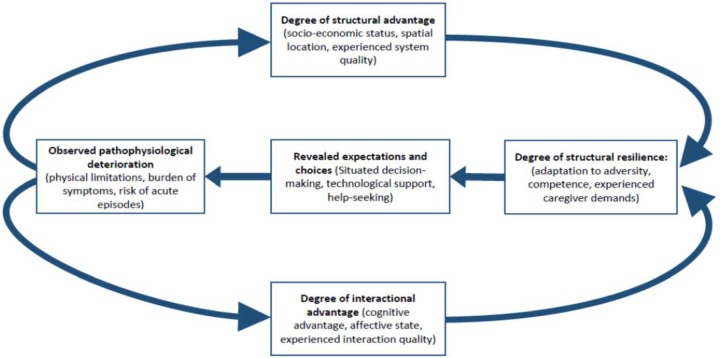
Factors that shape patient and caregiver expectations and choice.

An important secondary aim of this synthesis was to identify potential targets for interventions. In table 4, we identify and characterise a set of constructs that form a framework for the development of coordinated interventions that could act together to relieve the weight of structural, systemic, relational and individual disadvantages that are conferred on patients and caregivers experiencing these diseases and their comorbidities. Reducing these burdens is likely to improve patient outcomes and thus reduce system costs. However, it makes no sense to isolate them from each other. The constructs that are defined in table 4 represent phenomena that already are, or are in principle, measurable. In figure 3, we offer a logic model of the processes that link them: proposing that patient expectations and choices in the face of pathophysiological deterioration are mediated by their personal attributes (eg, socioeconomic status) as well as their experiences of healthcare, and moderated by the extent of resilience that they together with their caregivers and wider social support networks possess which may be influenced by a myriad of circumstantial factors.^25^ We need to better understand the design and implementation of interventions that might mitigate some of the key system and behavioural factors that negatively affect patient and caregiver experiences and outcomes, and that modify the chains of causation that are implicated in them.

**Table 4:** Factors affecting patient and caregiver experience of long-term life-limiting conditions

Finding	Measurable construct	Summary results of included papers: factors that shape patient experience of long-term life-limiting conditions
1. Structural, spatial and systemic disadvantages are important factors that inhibit active engagement with formal healthcare and self-management	Socioeconomic status	Patient experience is negatively affected by inequalities related to income;^62^ ^71^ age and gender;^39^ ^50^ ^54^ ^88^ and ethnicity^42^
	Spatial location	Patients and caregiver experience is negatively affected by unequal access to services and transport,^40^ ^70^ ^73^ ^76^ and unequal distribution of environmental pollution^40^ ^70^ ^73^ ^76^
	System quality	Patients and caregiver experience is negatively affected by poor professional support and material assistance,^40^ ^70^ continuity of care,^76^ coordination of services^43^ ^45^ ^48^ ^52^ and intraprofessional communications.^40^ ^45^ ^47^ ^48^ ^52^ Limited professional expertise in multimorbidities,^46^ and slow professional responses to anxieties and emergencies^76^ are also markers of poor system quality
2. Patients and caregivers experience multiple affective, cognitive and interactional disadvantages as they seek to participate in encounters with clinicians, decisions about their formal healthcare and self-management processes	Cognitive advantage	Patients and caregivers are cognitively disadvantaged by lack of educational resources^57^ ^83^ and information.^78^ ^81^ Disadvantage is exacerbated by poor understanding of disease and disease progression.^40^ ^41^ ^43^ ^45^ ^46^ ^50^ ^52^ ^56^ ^58^ ^59^ ^64^ ^69^ ^75^ ^76^ ^88^ These may lead to hypervigilance about symptoms, poor or confused symptom recognition,^43^ ^45^ ^57^ ^64^ ^88^ and ill-preparedness for crises^45^
	Affective state	Patients and caregivers may experience changes in self-identity, along with reduced self-esteem and self-worth, and loss of social functioning.^41^ ^43^ ^50^ ^54^ ^60^ ^79^ ^84^ ^89^ They may experience increased fear, anxiety, isolation and discomfort,^49^ ^51^ ^57^ ^60^ ^70^ ^82^ and this may lead to inappropriate responses to acute episodes^42^ ^47^ ^57^ ^67^ ^71^ ^74^
	Interaction quality	Good professional–patient relations,^42^ ^47^ ^76^ and individualised timing and type of information delivery^44^ ^53^ ^78^ ^83^ can have a positive effect on patient and caregiver experience. Fear of death^43^ ^50^ ^66^ ^80^ ^87^ negatively affects patient–professional interaction
3. Patients and caregivers value resilience, functional performance and social support that make a practical contribution to formal healthcare and self-management	Adaptation to disruption	Patient and caregiver experience is positively affected by adaptive processes;^43^ ^61^ ^63^ the normalisation of experienced symptoms and physical limitations^53^ ^71^ ^80^ and normalisation of self-management strategies.^63^ Patient and caregiver resilience,^58^ ^61^ ^73^ ^84^ capacity to manage uncertainty,^39^ ^42^ ^76^ ^82^ tolerance of disruption of everyday activities and competing clinical priorities^43^ ^45^ ^46^ ^55^ ^58^ ^87^ also positively affect patients experience
	Caregiver support	Caregiver support is defined by material assistance,^38^ ^55^ ^71^ ^84^ symptom management and self-management^57^ ^61^ ^65^ ^66^ and emotional and relational solidarity.^47^ It is negatively affected by perceived burdens and workload that interfere with normal life^38^ ^39^ ^76^ ^77^ ^81^ ^89^ ^90^
	Competence	Patients and caregivers demonstrate competence when they are able to exert control over disease progression,^74^ effectively participate in self-management,^77^ understand multimorbidities^46^ ^70^ and manage polypharmacy^64^
	Help seeking	Patient and caregiver help seeking is governed by interactions between expectations of clinical interventions,^76^ isolation and help seeking.^51^ ^64^ ^67^ ^70^ Help seeking is framed mainly through rational responses to emergency situations,^73^ and patients and caregivers valued the security and safety of hospital care^67^ ^73^
	Technological support	Technological interventions as life sustaining;^45^ ^73^ as mastery;^84^ ^85^ ^90^ as burdens;^58^ ^65^ ^79^ as intrusions;^81^ ^90^ risks of treatment failure/complications^85^ ^90^
	Situated decision-making	Patient and caregiver decision-making about help seeking and use of services was framed by the degree of awareness and uncertainty about prognosis;^41^ ^44^ ^48^ ^75^ ^83^ Difficulty and conflict over decisions^45^ ^86^ could be ameliorated using tools and techniques to increase patient control^77^ ^81–83^

### Limitations

Like all evidence syntheses, ours is subject to a number of important limitations. This is an overview of a heterogeneous set of reports. There was considerable variability in research aims, perspectives and methods. There were also very significant differences in authors' underlying assumptions about patient and professional motivation and behaviour. A limitation of syntheses of qualitative work is that these variations in assumptions and perspectives, and methodological heterogeneity, are often difficult to tease out. It is therefore not clear to what extent reviews continuously reproduce a particular set of ideas about why and how patient experiences and behaviours are a problem. Using an analytic approach that focuses on identifying, characterising and explaining sets of attributions brings these factors into the foreground and helps us to detect underlying conventional assumptions. We also identified all of the underlying qualitative studies covered by these reviews. It was clear that some reviews had interrogated very similar sets of primary research papers. This may also introduce problems of publication bias.

### Results in context

To the best of our knowledge, this is the first synthesis of qualitative systematic reviews focusing on patient experience of life-limiting chronic illness. Previous primary qualitative systematic reviews about the experiences of treatment burden in those with stroke and CHF excluded carer accounts and focused on developing taxonomies of treatment burden, excluding illness burden issues and did not seek to explain factors that shape patient journeys through care.^17^ ^52^ ^91^ We have explored long-term life-limiting conditions from the perspective of pathophysiological deterioration. Its point of departure was a consideration of patients' experiences of healthcare and burden of treatment in three long-term life-limiting conditions. This was situated against a background in which good decision-making about treatment, engagement with self-management and other treatment regimens, as well as conversations about end of life, are often regarded as problems of individual motivation and adherence. Many of the reviews included in this synthesis were aimed at informing professional practice and the development of interventions to support patients and caregivers with these illnesses. However, very few made concrete proposals in this regard. An analysis of this kind may be better placed to inform such work. A key finding of this synthesis is in its emphasis on what patients and caregivers value. This is not simply a matter of preferences. It also suggests that there are attributes of healthcare systems, relationships and practices that make experiences of pathophysiological deterioration worse.

## Conclusion

An important implication of this review is that patient and caregiver expectations and choices are not random or arbitrary but are the outcome of an experiential process. A paradigm shift is called for in service development and research in this area. Interventions that seek to empower individual patients may have limited effectiveness for those who are most affected by the combined weight of structural, interactional and resilience factors identified in this synthesis. There are likely causal interactions between these different factors that need to be better characterised and understood, but linking service and intervention development with robust theoretical models and rigorous practically oriented research ought to be the strategic direction for this. As a start in this direction, we have proposed domains of patient and caregiver experience that may represent good targets for new interventions that respond to the combined disadvantages that they may face.
