# Supplementary material for: Experiences of long-term life-limiting conditions among patients and carers: what can we learn from a meta-review of systematic reviews of qualitative studies of chronic heart failure, chronic obstructive pulmonary disease and chronic kidney disease?
Source: BMJ Open. 2016 Oct 5;6(10):e011694. doi: 10.1136/bmjopen-2016-011694 (PMC5073552; doi:10.1136/bmjopen-2016-011694)
Supplement: supplementary appendix [file bmjopen-2016-011694supp_appendix.pdf]

Appendix 1: Quality Appraisal Instrument

Reference (condition) :				
	Reviewer 1:	Reviewer 2:	Total Score	Total % for reference
Criterion	Reviewer 1 (Y/N/ don't know)	Reviewer 2 (Y/N/ don't know)		
1. Are qualitative methods appropriate to this synthesis?				
2. Are the aims of the synthesis clearly stated?				
3. Was a comprehensive literature search performed?				
4. Was the status of the publication (journal article, book chapter, thesis) used as an inclusion criterion?				
5. Was the scientific quality of the included studies assessed and documented?				
6. Was study selection and data extraction performed by more than one person?				
7. Were the methods for combining the results of studies appropriate?				
8. Is the analysis of data in the synthesis rigorous?				
9. Do the conclusions made, reflect the data presented?				
